# Treatment effectiveness of a mindfulness-based inpatient group psychotherapy in adolescent substance use disorder - study protocol for a randomized controlled trial

**DOI:** 10.1186/s13063-018-3048-y

**Published:** 2018-12-27

**Authors:** Christiane Baldus, Laura Mokros, Anne Daubmann, Nicolas Arnaud, Martin Holtmann, Rainer Thomasius, Tanja Legenbauer

**Affiliations:** 1 German Centre for Addiction Research in Childhood and Adolescence, University Medical Centre Hamburg-Eppendorf, Hamburg, Germany; 2 Landschaftsverband Westfalen-Lippe (LWL) University Hospital Hamm for Child and Adolescent Psychiatry, Ruhr-University Bochum, Hamm, Germany; 3 Department of Medical Biometry and Epidemiology, University Medical Centre Hamburg-Eppendorf, Hamburg, Germany

**Keywords:** Substance use disorder, Adolescents, Mindfulness-based intervention, Group psychotherapy, Child and adolescent psychiatry and psychotherapy

## Abstract

**Background:**

Current treatments for adolescents with substance use disorder (SUD) have had only limited success. In recent years, research has underlined the role of self-regulatory processes and impulsivity in the development and maintenance of SUD in adolescents. Mindfulness has gained much attention due to its capacity to influence self-regulatory processes, particularly in adult populations. Initial studies have shown the potential of mindfulness-based approaches in younger SUD patients. The aim of the present clinical trial is to evaluate the added treatment effect of a mindfulness-based group psychotherapy (“Mind it!”) for adolescents with SUD in comparison to the current standard treatment. Moreover, we seek to explore the feasibility of the intervention and possible mediators of treatment effects.

**Methods/design:**

There will be *N* = 340 participants aged between 13 and 19 years who are receiving child or adolescent psychiatric or psychotherapeutic inpatient or day treatment targeting their SUD and who have reported substance use 30 days before detoxification and do not show acute psychotic or suicidal symptoms at baseline. The study is a prospective randomized controlled multi-center trial in which patients are assessed: (1) after completing a prior detoxification phase (*t*_0_), (2) at 4 weeks (*t*_1_), (3) at 8 weeks (*t*_2_), and (4) at 6 months after *t*_2_ (*t*_3_). Participants in the intervention group will receive mindfulness-based group psychotherapy in addition to their existing treatment regime. The primary outcome is substance use in the past 30 days at follow-up based on the Timeline Followback self-report. Secondary outcomes include craving, severity of dependence, and abstinence motivation. Mindfulness, impulsivity, and emotion regulation will be analyzed as possible mediators of treatment effects.

**Discussion:**

This trial is expected to provide evidence of the added effect of a novel, safe, and feasible treatment option for adolescents with SUD.

**Trial registration:**

German Register of Clinical Studies, DRKS00014041. Registered on 17 April 2018.

## Background

The use of alcohol and illegal drugs and their associated detrimental health outcomes amount to 19.737 disability adjusted life years (DALYs) in Europe [1]. Adolescence is a vulnerable period for the development of substance use disorders (SUDs). Specifically, when individual substance use patterns exacerbate, the risks for negative health and psychosocial outcomes increase [2, 3]. Prevalence rates of SUDs in children and adolescents from population-based samples have not yet been measured in Germany, but some previous research allows for estimates: 10 to 15% of German males aged 14–24 were found to have alcohol dependence or misuse according to DSM-IV criteria. Cannabis abuse was reported by 5.5% and cannabis dependence by 2.2% in the same sample [4]. National health report data show that 34% of patients with substance dependence are younger than 25 years [5] and about 35,000 patients under 20 years receive costly inpatient treatment due to diagnoses relating to “mental or behavioral disorders due to psychoactive substance use” (ICD-10, chapter F10-F19). In essence, SUDs in children and adolescents are an enormous public health burden.

Although progress has been made for both access to and availability of pharmacological and psychotherapeutic SUD treatments [6, 7], current research on the effects of treatment in SUD patients for various age groups shows disappointing results. Relapse rates range between 50% and 81% across various delivery modes, such as highly structured outpatient settings [8], inpatient settings [9], and combined psychotherapeutic and pharmacological treatments [10]. Specific research on SUD patients who are minors is scarce [11], but would be greatly desirable [7].

In recent years, there has been significant research on (neuro-)biological mechanisms for the development of substance use in minors. The research into mechanisms focuses on self-regulatory processes, specifically, motivational aspects such as reward responsiveness, delay discounting, and impulsivity [12, 13]. However, the findings on SUD mechanisms have barely been translated into treatment programs, which may be a reason for the limited success of SUD treatment in minors [6, 13–16].

Preliminary evidence from research-informed treatments links self-regulatory models of SUD to mindfulness-based approaches, because mindfulness targets several aspects of self-regulation [6, 12, 13, 17]. Mindfulness is defined as bringing full awareness to present-moment experiences in an accepting, non-judgmental, and open-minded way [18]. Mindfulness skills are associated with cognitive and affective stability and flexibility, adaptive coping, and reduced cue-reactivity towards stress-induced cravings [12, 19]. Initial neuroimaging [19, 20] and clinical studies [21] provide evidence that mindfulness skills can help SUD patients. Mindfulness brings attention to highly automated and minimally controlled habits, which are often involved in craving and substance use relapse. Mindfulness practice can help people to become aware of craving and substance use habits. This opens opportunities for modifying reactions instead of following established stimulus–response behaviors, which may involve modifying unpleasant sensations or emotions with substance use. This could reduce the risk of relapse in SUD patients.

Sanger and Dorjee [22] produced strong neuropsychological evidence for the positive effects of mindfulness practice in adolescence. Affective self-regulation and coping skills were enhanced by changes in prefrontal brain functioning. The current literature suggests that mindfulness practice impacts affective stability and attentional control and may, therefore, be useful in SUD treatment [23].

The effects of mindfulness-based interventions in adult clinical populations are well documented. Mindfulness-based interventions target different patient groups, such as mindfulness-based stress reduction for chronically ill patients [24, 25], mindfulness-based cognitive therapy (MBCT) for depressed patients [26] and mindfulness-based relapse prevention (MBRP) for SUD patients [27–29]. Even in comparison to active control groups, these interventions were able to reduce legal and illegal substance use [30–32], cravings [13, 17], and relapse rates [30–32]. Up to now, no unwanted side effects of mindfulness-based interventions have been reported for relevant target groups.

Clinical evidence for underage patient groups is scarce. Several clinical studies focus solely on specific clinical aspects of mindfulness-based interventions such as outpatient aftercare [30] or sleep improvement [33]. One work focuses on co-occurring post-traumatic stress disorder and addiction [34]. Another study was a qualitative pilot study, which limits its generalizability [35]. Evidence for the use of mindfulness-based interventions for child and adolescent substance use is not as clear as in adult patients [36–38]. However, initial results show that mindfulness practice helped to complement cognitive behavior therapy approaches in children with emotion regulation deficits [39].

According to current evidence, mindfulness-based interventions are safe and effective. Mindfulness practice can help to improve attentional control, depressive symptoms, anxiety, rumination, externalizing problem behavior, social skills, and stress. Moreover, SUD-specific problems, such as emotional self-regulation and craving, have been shown to improve through mindfulness-based approaches. As yet, there are no mindfulness-based treatments for younger SUD patients that have adapted tested mindfulness-based SUD approaches in a developmentally appropriate way. There is a need for high-quality clinical trials that focus on the reduction of substance use as the primary outcome to evaluate the full potential of mindfulness-based treatment approaches in underage SUD patients. To our knowledge, there have been no such randomized controlled clinical trials.

With the current study, we aim to answer the following two questions:

1. Is there an additive treatment effect for young SUD patients who receive complementary mindfulness-based group therapy, as shown by higher 30-day abstinence rates 6 months after treatment, in comparison to participants receiving standard youth psychiatric SUD inpatient treatment? In secondary exploratory analyses, we want to see if the effects of the mindfulness-based group therapy on treatment are mediated through factors that are related to the content of the intervention, such as impulsivity, (trait-)mindfulness, emotion regulation, and perceived stress.
2. Can a mindfulness-based treatment manual for SUD adolescents be implemented and used to support and complement current SUD treatments effectively in inpatient youth psychiatric settings?

## Methods/design

### Design

The current trial is a subproject run by the consortium IMAC-Mind (Improving Mental Health and Reducing Addiction in Childhood and Adolescence through Mindfulness: Mechanisms, Prevention and Treatment), which focuses on the use of mindfulness-based approaches for different SUD populations. The current study is a prospective randomized controlled trial with treatment as usual for the control group.

### Sample

Study participants will be patients diagnosed with a SUD (ICD-10: F10-F19) aged 13 to 19 years who are currently receiving inpatient or daycare clinical treatment for that condition and who have completed detoxification. Subjects are diagnosed by treating clinicians and their diagnoses are verified in additional clinical interviews by study personnel. Patients will be excluded from the study if they show symptoms of acute suicidality, schizophrenia, or other disorders with acute psychotic symptoms. Further exclusion criteria are an IQ under 70, insufficient knowledge of German by either patients or their parents or guardians, and no substance use in the 30 days prior to detoxification. Patients’ medication is not an exclusion criterion but will be carefully documented.

### Measurement

**Fig. 1:**
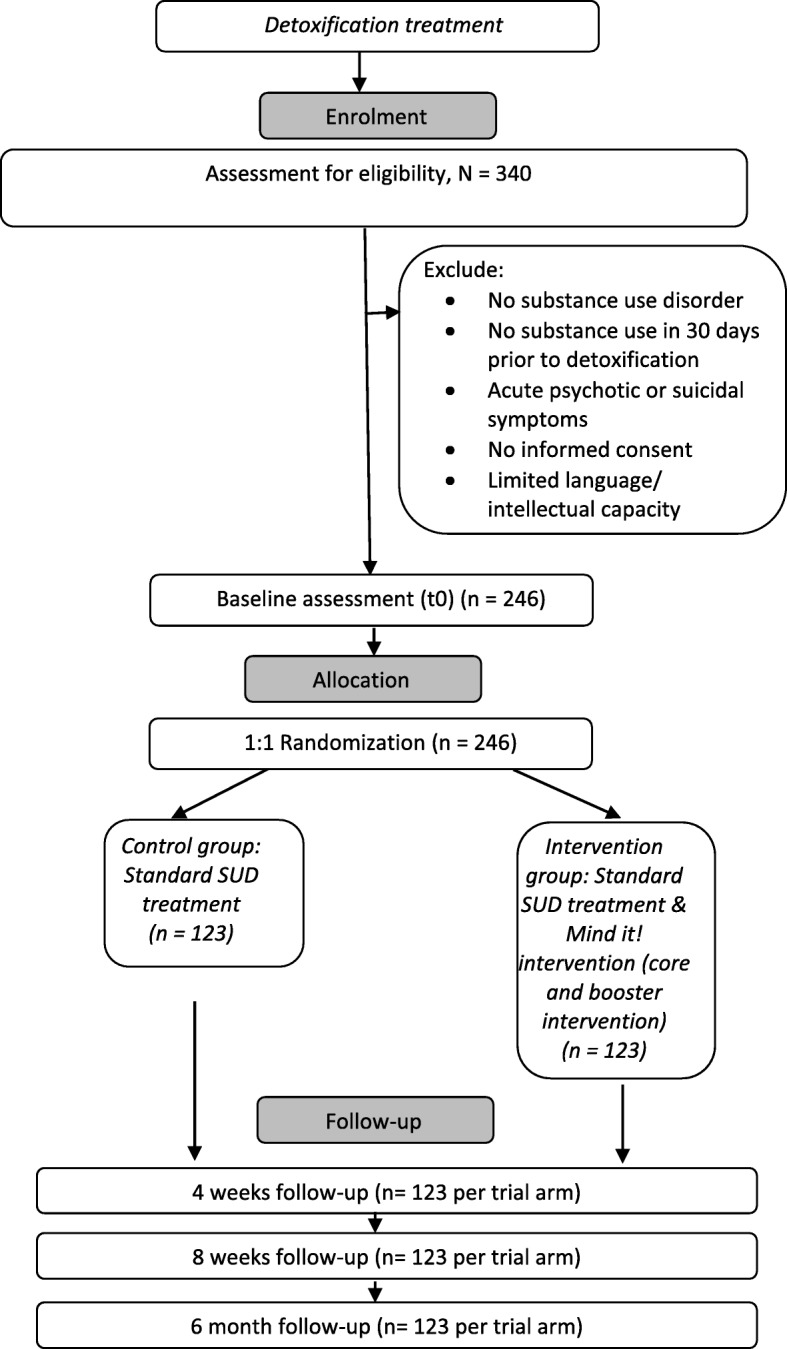
Flow diagram for the Mind it! trial. SUD Substance use disorder

Participants are asked questions in structured clinical interviews and they complete questionnaires. Their hands are scanned so that we can calculate the 2D:4D ratio (*t*_0_ only), and they undergo a computerized assessment for several neuropsychological tasks at four points within the study process: after detoxification and before randomization and the following SUD treatment phase (*t*_0_), 4 weeks into treatment (*t*_1_), 8 weeks after *t*_0_ (*t*_2_), and 6 months after *t*_2_ (*t*_3_). Figure 1 gives an overview of the measures used.

### Methods against bias

The study follows the CONSORT statement for randomized controlled trials [40]. This includes the publication of a study protocol and the study’s public registration (German Register of Clinical Studies, DRKS00014041). The procedures for study inclusion and assessment (obtaining informed consent, handling of questionnaires, clinical records, and clinical interviews) are performed by study personnel who remain blind to the participants’ randomization to either the intervention group (mindfulness-based group intervention “Mind it!”) or the control group (treatment as usual) and who are not involved in providing treatment. Blinding of patients is not possible due to their (non-)participation in the mindfulness-based group intervention. Self-reports regarding substance use are validated through urinalyses, which are routinely taken as part of SUD treatment and are extracted from medical records. Diagnostic assessments are based on highly standardized measurement tools and clinical information is provided by experienced clinicians. All study personnel are trained to conform to current guidelines and regulations (e.g., data safety). Questionnaire data will be computerized by trained staff using the EpiData software to ensure the high quality of the entered data. Additionally, a clinical research organization will monitor adherence to the study protocol and overall study quality. A data and safety monitoring board will oversee study procedures, recruitment, and data flow twice yearly and advise project staff when necessary.

### Procedure

The procedures in the current study have been approved by the ethics board of Ruhr University Bochum (176268). During participant recruitment and treatment, two study centers (Hamm and Hamburg; Hamm is the coordinating center) will provide a mindfulness-based group intervention in addition to what is routinely provided as standard inpatient SUD treatment. Patients admitted to either study center because of SUD treatment and who have completed detoxification are given information about the study and their possible participation if they are eligible for study participation according to the inclusion and exclusion criteria. After written informed consent is given by both the patient and their parent or guardian, the *t*_0_ assessment will be performed within 1 week after the transition from detoxification treatment to after-detoxification treatment. After the *t*_0_ assessment, patients will be randomized to either the intervention group (mindfulness-based group intervention in addition to standard treatment) or the control group (standard treatment as usual). The patients will be randomly allocated to either the Mind It! group or the control group in a 1:1 allocation ratio, stratified by study center with variable block lengths. The randomization lists will be prepared by the Department of Medical Biometry and Epidemiology of the University Medical Center Hamburg-Eppendorf. The central allocation procedure is managed by the coordinating study center in Hamm to guarantee allocation concealment.

**Fig. 2:**
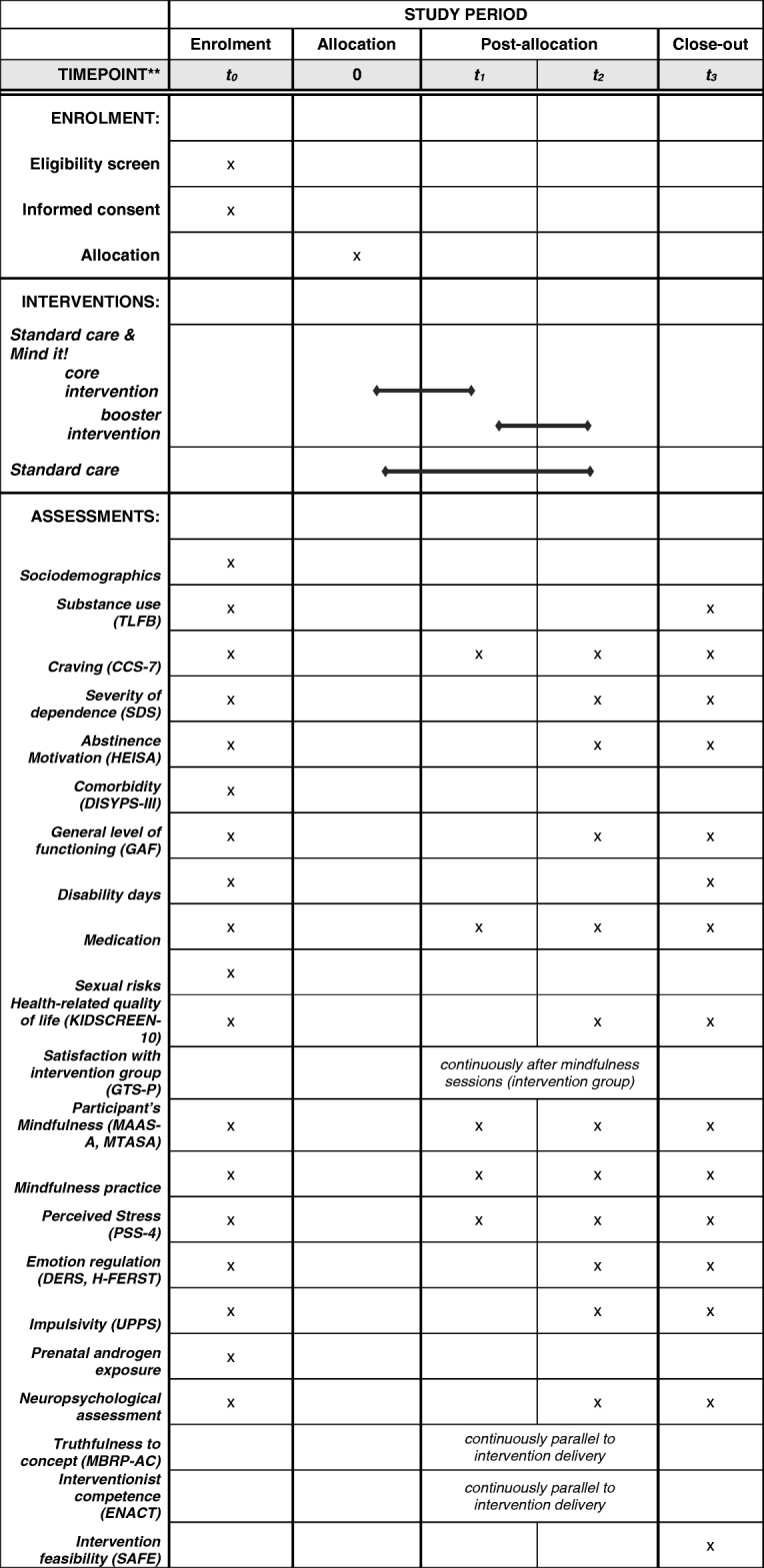
Schedule of enrolment, interventions, and assessment for the Mind it! trial. CCS-7 cannabis craving screening, DERS Difficulties in Emotion Regulation Scale, ENACT Enhancing Assessment of Common Therapeutic Factors, GAF Global Assessment of Functioning, GTS-P Gruppentherapiestundenbogen, HEISA Heidelberg scale for abstinence confidence, H-FERST Heidelberger Fragebogen zur Erfassung von Emotionsregulationsstrategien, MAAS-A Mindful Attention Awareness Scale for Adolescents, MBRP-AC Mindfulness Based Relapse Prevention Adherence and Competence Scale, MTASA Mindfulness Thinking and Action Scale for Adolescents, PSS-4 Perceived Stress Scale, SAFE Structured Assessment of Feasibility, SDS Severity of Dependence Scale, TLFB Timeline Followback, UPPS impulsive behavior scale

The intervention group receives a mindfulness-based group intervention consisting of 12 45-min sessions relating mindfulness techniques to substance use problems in adolescents. In each of the first 4 weeks of the intervention, the participants receive two 45-min sessions of the core mindfulness-based group intervention. At the end of the 4 weeks, they undergo the first interim assessment (*t*_1_). In weeks 5–8 of treatment, intervention group participants receive one weekly 45-min session of the sustained mindfulness-based group intervention (maximum of four sessions). After the total mindfulness-based group intervention and after 8 weeks of treatment, participants undergo another assessment (*t*_2_), which is followed by the final assessment (*t*_3_) 6 months after *t*_2_. Assessments for *t*_3_ are performed by study personnel and participants receive €50 and travel expenses as compensation for participation in the *t*_3_ assessment. The mindfulness-based group intervention is delivered by clinically experienced researchers, who are currently in therapeutic training or have finalized therapeutic training, and are experienced in giving mindfulness-based interventions. Figure 2 gives an overview of the study flow.

### Standard SUD treatment

In both study centers (Hamm and Hamburg), the standard SUD treatment has two structured, multidimensional treatment phases: (1) During the detoxification phase of treatment, the protected environment guarantees safe detoxification and constant monitoring of possible withdrawal symptoms. Within that setting, detailed psychiatric, neuropsychological, and psychosocial diagnostic assessments are performed. First psycho-education elements are used to address patients’ background SUD problems. Moreover, patients are motivated to continue treatment after detoxification. (2) Treatment after the detoxification phase addresses comorbid problems and disorders as the background of their SUD. Psycho-educational interventions are used to motivate patients to abstain from substance use in their future life and they are taught useful skills (e.g., against relapse). The SUD treatment integrates psychotherapeutic, somatic, and medical interventions with kinesiatrics, family therapy, occupational and educational therapy, and music therapy. These treatment elements are delivered in individual or group sessions.

### Intervention group

The mindfulness-based group intervention under evaluation in this study will be written up as a manual and is based on previous studies on MBCT [26, 41], MBCT for children [42], and MBRP [26–30, 41]. Research has indicated that MBRP can significantly reduce the risk of craving-induced relapses in SUD patients. The current manual will be closely aligned to the existing English-language treatment manual by Himelstein and Saul [43], *Mindfulness-based substance abuse treatment for adolescents*, which was developed in California for incarcerated youth with SUD problems but was later validated in inpatient, outpatient, educational, and community settings as a group intervention [35, 43–45].

The mindfulness-based group intervention integrates both formal mindfulness meditation practices from MBCT (such as body scan meditation, mindfulness of the breath meditation, mindfulness of the body in movement, and sitting meditation) and informal mindfulness practices, which supports participants to integrate mindful awareness into their daily routines. Inquiry is used as a group technique to reflect on experiences during meditation and psycho-education elements are used to teach participants about substance use, stress, stress reactivity, cravings, and coping skills, and they are given a rationale on why mindfulness helps with substance use problems. Each session includes formal mindfulness meditation practice and participants are encouraged to practice mindfulness outside the group sessions.

The group intervention mindfulness practice is adapted to the developmental needs of the young participants regarding duration and content. Therefore, we decided to make group sessions rather short (45 min) but deliver them more often compared with mindfulness-based intervention sessions with adults, such as, for example, MBCT or MBRP. Group sizes will be limited to a maximum of eight participants, so that individual attention can be given to them. Moreover, we plan to integrate simple sensory exercises, movement exercises, and playful activities with significant variation in the mindfulness practice rather than delivering an extended talk. Sessions will include SUD-specific treatment elements from MBRP (such as the identification of possible relapse triggers), mindfulness-based coping skills (such as acceptance), and skills specifically targeting cravings (such as urge surfing) [30].

The first eight sessions of the intervention (the core intervention) focus on delivering this content. The final four sessions (the sustained intervention) extend on this in that the participants are strongly encouraged to practice meditation and mindfulness every day using audio material and written intervention material and to record their practice on diary cards.

### Primary outcome

Participants’ substance use will be assessed using the standardized, calendar-based Timeline Followback (TLFB) interview format [46]. In this interview, we will use paper-based screening questions on lifetime use of nicotine, alcohol, cannabis, opioids, cocaine/ crack, designer drugs, methamphetamine, amphetamine, unprescribed medication, and other drugs so that we can target only those substances that each patient has used. The TLFB interviews will also focus on substance use within the past 30 days prior to detoxification and assessment (*t*_0_). The primary outcome of the study is the number of days with substance use within the past 30 days 6 months after the end of the intervention (*t*_3_) [32, 47].

### Secondary outcomes and mediating variables

Secondary outcomes include: (a) measures associated with SUD symptoms such as cravings, severity of dependence, relapses, and abstinence motivation, (b) measures of comorbid symptoms and overall functioning, such as general level of functioning, number of disability days (clinical standard), current medication (clinical standard), sexual risks, and health-related quality of life, (c) participant satisfaction with the mindfulness-based group intervention (intervention group only), and (d) proximal measures relating to intervention content such as mindfulness, mindfulness practice, perceived stress, emotion regulation, and impulsivity. For this last category of secondary outcomes (d), we also seek to explore whether changes in these measures possibly mediate the effects of the mindfulness-based group intervention on SUD symptoms, such as cravings or substance use.

#### Cravings

The inventory for an assessment of cannabis cravings (CCS-7) [48] is a validated German seven-item version of the English language Marijuana Craving Questionnaire [49]. The CCS-7 comprises two dimensions of cravings: relief cravings (e.g., “If I were smoking marijuana right now, I would feel less nervous”) and reward cravings (e.g., “It would be great smoking marijuana right now”), with Cronbach’s alpha ranging from .78 to .85. For participants who do not identify cannabis as a problem substance, we provide visual analogue scales to measure their current cravings for problem substances.

#### Severity of dependence

The Severity of Dependence Scale (SDS) [50] was developed to assess the psychological components of substance dependence symptoms. It comprises five items and focuses on symptoms of loss of control and distress with regards to substance use (e.g., “Did you think your use of … was out of control?”). This scale has good validity and internal consistency (Cronbach’s alpha ranging from .81 to .90).

#### Abstinence motivation

The Heidelberg scale for abstinence confidence (HEISA) [51] is a German self-report measure that assesses patients’ confidence in whether they can abstain from substance use in different situations. The introductory item of this scale assesses patient’s goal with regards to their future substance use, asking respondents to choose their substance use goals ranging from “remain totally ‘clean’ – never use substances again” to “use substances as before.” We use this single introductory item to assess each participant’s abstinence motivation .

#### Comorbidity

Comorbid disorders will be assessed through clinical interviewing and the DISYPS-III diagnosis checklists on symptoms of attention deficit and hyperactivity disorder, conduct disorder, depression, anxiety, and trauma [52]. The checklists are used to diagnose disorders within these symptom spectra according to ICD-10 and DSM-V.

#### General level of functioning

We will use the ICD-10 Global Assessment of Functioning (GAF) to assess participants’ social and psychological functioning on a numeric scale (1 = persistent danger to 100 = no symptoms). The assessment will be carried out by clinicians or trained study personnel.

#### Number of disability days

Within our clinical standard procedure, we assess the number of days within the past 30 days on which participants felt unfit to attend school or pursue their normal daily activities.

#### Medication

As a further clinical standard procedure, we will document any medication use from 30 days before treatment begin to the *t*_3_ assessment.

#### Sexual risks

As a further indicator of previous trauma and sexual health behavior, we ask participants to indicate previous incidents of sexual harassment and sexual assault. These items have been used successfully in previous research [53, 54].

#### Health-related quality of life

KIDSCREEN-10 is a 10-item validated and reliable self-report tool for the assessment of subjective health and well-being (e.g., “Do you feel fit and well?”) in children and young people aged up to 18 [55]. Cronbach’s alpha for KIDSCREEN-10 is .82.

#### Satisfaction with intervention group

Patients will be asked to provide self-reports of how they viewed the mindfulness-based group intervention through the *Gruppentherapiestundenbogen* (GTS-P) [56], an eight-item German-language tool (e.g., “The atmosphere within the group was good today”), which has good validity and internal consistency (Cronbach’s alpha between .79 and .91).

#### Participant’s mindfulness

Due to the centrality of mindfulness in our study, mindfulness will be measured through two self-report assessment tools: (1) the Mindful Attention Awareness Scale for Adolescents (MAAS-A) [57], which was chosen by the larger consortium into which our study is embedded, and (2) the Mindfulness Thinking and Action Scale for Adolescents (MTASA) [58], which has proved to be valuable in previous research on the intervention [44]. MAAS-A is a 14-item scale that mostly targets the absence of mindful experiences (e.g., “I snack without being aware that I’m eating”). It has shown good internal consistency (Cronbach’s alpha from .82 to .84) and validity in both normative and psychiatric samples and has been proved to be sensitive to change in mindfulness-based intervention research. MTASA is a 32-item scale and consists of four subscales that focus on different aspects of mindfulness: healthy self-regulation, active attention, awareness and observation, and accepting experiences. MTASA has been validated and both the total scale (Cronbach’s alpha = .86) and the subscales (Cronbach’s alpha between .63 and .85) have acceptable internal consistency.

#### Mindfulness practice

Participants will be asked to report their previous experiences with mindfulness-related practices (e.g., meditation and yoga) as well as their ongoing mindfulness practices (both formal mindfulness practices, e.g., meditation on deep breathing, and informal mindfulness practices) in an interview using the TLFB format. This procedure has been used in previous mindfulness-based intervention studies [47].

#### Perceived stress

The Perceived Stress Scale (PSS-4) [9, 59, 60] is a validated and economic four-item self-report scale to measure perceived stress. The items focus on feelings of subjective control or being overburdened (e.g., “In the last month, how often have you felt difficulties were piling up so high that you could not overcome them?”).

#### Emotion regulation

Participants’ emotion regulation will be measured by two assessment tools, the Difficulties in Emotion Regulation Scale (DERS) [61] and *Heidelberger Fragebogen zur Erfassung von Emotionsregulationsstrategien* (H-FERST) [62]. DERS is a 36-item measure. It has been validated in an adolescent community sample and its internal consistency is good. It consists of six subscales (strategies, non-acceptance, impulse, goals, awareness, and clarity) and has shown good internal consistency (Cronbach alpha between .76 and .89) in community samples. H-FERST is a more clinically oriented 39-item self-report measure, which showed good reliability. It consists of nine subscales: rumination, reappraisal, acceptance, problem-solving, suppression of emotional expression, suppression of emotional experience, avoidance, activity/social support, and distraction.

#### Impulsivity

We will use the UPPS impulsive behavior scale as a measure of impulsivity [63], UPPS is an acronym for the scale's targeted concepts urgency, (lack of) premeditation, (lack of) perseverance, and sensation seeking [64]. It has good internal consistency (Cronbach’s alpha = .87) and has been validated.

### Further measures for wider use in the consortium

Several assessments integrated into this study will be used to answer the research questions of the whole IMAC-Mind consortium.

#### Prenatal androgen exposure

Maternal stress and substance use during pregnancy are associated with increased prenatal sex hormone exposure, which is linked to reduced self-regulation and increased psychiatric problems and SUDs in the resulting offspring [65–68]. Prenatal assessment of androgen exposure is difficult, but in various studies, the ratio of the lengths of the second and fourth fingers (2D:4D) has successfully been used as a proxy of intrauterine exposure to gonadal steroids. A low 2D:4D ratio indicates a higher level of prenatal androgen exposure [69] and lower self-regulatory competence in later life. The 2D:4D ratios of the participants, therefore, will be used as a proxy of the adolescents’ disposition to self-regulatory competences, which in turn are associated with SUDs. Participants’ hands will be scanned, and their finger lengths will be measured to obtain the 2D:4D finger length ratio.

#### Neuropsychological testing

A neuropsychological test battery will be integrated into the study to measure participants’ impulsivity, decision-making, reward processing, and risk-taking behavior, all of which have been linked to substance use in adolescents [70]. Three established tasks will be applied: a monetary incentive delay task [71], a stop signal task [72], and the Cambridge gambling task [70].

### Adherence to mindfulness concepts and the quality of intervention delivery

We aim to record sessions on video and evaluate a proportion of mindfulness-based group intervention sessions, from which randomly chosen 20-min intervals are drawn to assess the quality of intervention delivery. Two target variables are assessed independently using the videos: adherence to the standard for mindfulness-based interventions for SUD patients and the competence of the interventionist.

Fidelity to the mindfulness-based interventions will be rated on the Mindfulness Based Relapse Prevention Adherence and Competence Scale (MBRP-AC), which was originally designed for the MBRP program developed by Chawla and colleagues [73]. It has good validity and inter-rater reliability. As our current intervention resembles that program, we felt the items were suitable for the context of this study, too.

The competence of the interventionist, which is independent of mindfulness, is rated with items 1–5 of the Enhancing Assessment of Common Therapeutic Factors (ENACT) scale developed by Kohrt and colleagues [74]. The selected items assess the therapists’ communications skills, rapport building, and empathy. The scale assesses a therapist’s competence for supervision and training and showed good psychometric accuracy.

At least two researchers, with a background in mindfulness-based interventions and therapeutic competence, will independently rate the recorded sessions using both scales (MBRP-AC and ENACT). Inter-rater congruency will be calculated and documented. Each study center (Hamm and Hamburg) will continuously monitor intervention delivery in the other center using the video recordings.

### Intervention feasibility

The interventionists will assess their perception of the feasibility of the mindfulness-based group intervention using the Structured Assessment of Feasibility (SAFE) [75] after the final session. This scale has good inter-rater reliability (*k* = .84) and test–retest reliability (*k* = .89).

### Safety

A set of adverse events (AEs) and serious adverse events (SAEs) were defined and their prevalence and any possible causal relation to the new mindfulness-based group intervention under evaluation were tracked. All AEs and SAEs, from the first mindfulness-based group intervention to 28 days after the last mindfulness-based group intervention, will be documented in the patient’s file. Each AE and SAE will continue to be monitored until the event has terminated and the participant’s state has improved or at least proved to be stable but no longer than 3 months after the end of the participant’s treatment. The study team will evaluate any possible causal relationship between AEs and SAEs and participation in the mindfulness-based group intervention and documents this.

### Sample size calculation

Based on prior research [29, 31, 32], we expect to detect a small to medium-sized effect of *d* = 0.36 for our primary outcome. We calculated the sample size using G*Power [76], with a two-sided alpha level of 0.05 and a study power of 80%. The required sample will comprise 246 participants (123 both in the control group and in the intervention group). To achieve this sample size, we seek to screen *N* = 340 patients for eligibility for the trial (Fig. 1).

### Analyses

Data will be analyzed according to the CONSORT statement [40]. A detailed statistical analysis plan will be prepared and finalized before breaking the blind. Descriptive statistics will be determined for the intention-to-treat population according to treatment assignment. The intention-to-treat population consists of all randomized patients. The primary hypothesis is that the intervention is superior to treatment as usual according to the number of days with substance use within the past 30 days 6 months after the end of the intervention (*t*_3_). A mixed negative binomial regression will be performed based on the intention-to-treat population with treatment group, time, interaction between treatment group and time, and recruitment center as fixed effects, the number of days with substance use within the past 30 days at baseline as a covariate, and patient and therapy group as random effects. The contrast of the treatment group at *t*_3_ will be interpreted in a confirmatory manner. We will report adjusted group differences with corresponding 95% confidence intervals and *p* values. Mixed models are more robust against missing values than models without random factors and yield unbiased estimates under the missing at random assumption. For model diagnostics, a systematic examination of factors associated with loss to follow-up will be conducted. Missing values will be imputed in a sensitivity analysis by the multiple imputation method. Additionally, the primary analysis will be conducted on the per protocol population. The following subgroup analyses will be planned. The secondary outcomes will be analyzed in an exploratory manner with appropriate models with the same covariates and fixed and random effects as in the primary analysis. Multiple mediation models for identifying the mechanisms of intervention effects (with a focus on behavioral measurements of self-regulation and mindfulness) will be used [77]. The safety end points will be determined with a mixed logistic regression. Interim analyses are not planned. The two-sided type I error will be set at 5%. Statistical analyses will be carried out with SPSS, Version 21 (IBM Corp., Armonk, NY).

## Discussion

The aim of the proposed clinical trial is to investigate the added benefit of a mindfulness-based group intervention for young patients (aged 13–19 years) in inpatient or daycare treatment for SUD after a prior detoxification phase. Previous studies have shown that mindfulness-based interventions are safe and feasible for adolescent substance users [35, 44]. The current project will also investigate the feasibility of the intervention in a German psychiatric and psychotherapeutic medical treatment setting for children and adolescents. While we are interested in determining the clinical potential of such interventions in reducing substance use in a younger patient population, our assessments are designed to give insights into potential mechanisms of mindfulness-based interventions on substance use. Analyses of secondary outcomes such as perceived stress, mindfulness, impulsivity, and neuropsychological functioning will allow us to investigate the assumed mediating effects of these variables, several of which have been identified as possible factors linking mindfulness approaches with substance use problems [17]. Research questions relating to biological markers of self-regulation and the neuropsychological processes of mindfulness-based interventions in association with SUDs will be further investigated in associated projects run by the wider research consortium IMAC-Mind, of which the current project is a part.

We hope that the results of this study will be published in international research journals and conferences and provide further evidence for the use of mindfulness-based approaches for young people with SUDs. The current project is important as it is the first randomized controlled trial for this target group to focus on substance use outcomes. New approaches for young SUD patients are important. Until now, current treatment options for young SUD patients have had limited effects, but a wide range of research indicates the detrimental effect of prolonged substance use and SUDs on developmental outcomes for young people.

### Trial status

The trial is ongoing and currently recruiting.
